# Comparative Analysis of Normalised Difference Spectral Indices Derived from MODIS for Detecting Surface Water in Flooded Rice Cropping Systems

**DOI:** 10.1371/journal.pone.0088741

**Published:** 2014-02-20

**Authors:** Mirco Boschetti, Francesco Nutini, Giacinto Manfron, Pietro Alessandro Brivio, Andrew Nelson

**Affiliations:** 1 Institute for Electromagnetic Sensing of the Environment, National Research Council, Milan, Italy; 2 Department of Agricultural and Environmental Sciences – Production, Landscape, Agroenergy, University of Milan, Milan, Italy; 3 Social Sciences Division, International Rice Research Institute, Los Baños, Philippines; NASA Jet Propulsion Laboratory, United States of America

## Abstract

Identifying managed flooding in paddy fields is commonly used in remote sensing to detect rice. Such flooding, followed by rapid vegetation growth, is a reliable indicator to discriminate rice. Spectral indices (SIs) are often used to perform this task. However, little work has been done on determining which spectral combination in the form of Normalised Difference Spectral Indices (NDSIs) is most appropriate for surface water detection or which thresholds are most robust to separate water from other surfaces in operational contexts. To address this, we conducted analyses on satellite and field spectral data from an agronomic experiment as well as on real farming situations with different soil and plant conditions. Firstly, we review and select NDSIs proposed in the literature, including a new combination of visible and shortwave infrared bands. Secondly, we analyse spectroradiometric field data and satellite data to evaluate mixed pixel effects. Thirdly, we analyse MODIS data and Landsat data at four sites in Europe and Asia to assess NDSI performance in real-world conditions. Finally, we test the performance of the NDSIs on MODIS temporal profiles in the four sites. We also compared the NDSIs against a combined index previously used for agronomic flood detection. Analyses suggest that NDSIs using MODIS bands 4 and 7, 1 and 7, 4 and 6 or 1 and 6 perform best. A common threshold for each NDSI across all sites was more appropriate than locally adaptive thresholds. In general, NDSIs that use band 7 have a negligible increase in Commission Error over those that use band 6 but are more sensitive to water presence in mixed land cover conditions typical of moderate spatial resolution analyses. The best performing NDSI is comparable to the combined index but with less variability in performance across sites, suggesting a more succinct and robust flood detection method.

## Introduction

Timely and accurate information on crop typology and status is required to support suitable action to better manage agricultural production and reduce food insecurity [1], [2]. More specifically, spatial information on where, when and how staple crops are cultivated is an important input for spatialized crop growth models for yield forecasts and yield gap analyses [3], [4]. Digital cartographic data related to crops and croplands at global/regional scales (i.e. [2], [4]–[9]) represent a static or snapshot view of a strong dynamic process and although they are valuable and essential datasets they do not provide information to monitor and model the annual and seasonal changes in crop status and crop production which are prerequisites for monitoring food security. There is a recognised need to develop and test methods which can provide operational information for staple crops, including rice [10] and automated classification and detection techniques are vital to ensure sustainability and transparency.

We focus on rice for several reasons. Firstly, it is the world’s most important staple crop; it is the second largest in terms of harvested area after wheat, but is by far the most important in terms of human consumption, especially in low- and lower-middle-income countries (FAOSTAT, 2012). Thus, reliable and timely information on the global rice crop is required to guide food security and food price policies. Secondly, it truly is a global crop cultivated across a wide range of environmental conditions. Rainfall in rice-growing areas can vary from over 5,000 mm/y along Myanmar’s Arakan Coast to less than 100 mm in Al Hasa Oasis in Saudi Arabia; average temperature during the rice season can vary from highs of 33°C in Sindh, Pakistan, to lows of 17°C in northern Japan; and rice can be cultivated from sea level up to 2600 m on the terraces of Nepal and Bhutan [11]. Thirdly, rice is grown under a wide range of management conditions such as highly mechanised, irrigated, single summer cropping (i.e. Italy, Japan, the U.S., Australia, Brazil); the more marginal rainfed rice systems across Latin America, sub-Saharan Africa, and South and South-East Asia; in rotation with other crops such as the vast rice/wheat double-cropping areas of India and China; intensive, irrigated triple cropping (i.e. Indonesia, Vietnam); and various other double and triple mixed-cropping combinations across Asia, Africa and Latin America.

Distilling the results of the many studies on rice crop detection and characterisation into an operational system is clearly challenging due to the geographic extent of rice, the range of environments under which it is cultivated and the diversity of crop management methods. Rice has been well studied in the remote sensing literature, and several mapping methods have been proposed to identify rice cultivated area based on the agronomic practise of pre-season flooding which characterises the majority of rice cropping systems (namely, irrigated rice, lowland rainfed rice and deepwater rice) which account for over 90% of the 154 M ha [11] cultivated with rice each year.

The detection of this flooded condition at the start of the growing season, followed by a rapid increase in observed biomass, is the key element for discriminating a rice crop from other natural vegetated surfaces and/or other crops. Although assessments of different methods for detecting biomass and changes in biomass under different environments are abundant, there is no comparable body of research on methods for detecting agronomic flooding. A thorough investigation of methods to detect agronomic flooding would seem to be a prerequisite for developing an operational rice crop detection methodology. In this paper, we focus attention on robust and reliable normalised difference spectral indices (NDSIs) to detect agronomic flooding practises which are particular to rice.

### 1.1 Normalised Difference Spectral Indices in the Literature

NDSIs have been proposed as an operational tool to perform water surface detection in different contexts [12], [13] and NDSIs related to water surface presence and vegetation indices that monitor rice biomass growth have been proposed as part of a paddy rice detection algorithm [14]–[17]. In general, water-related indices are proposed as a combination of shortwave infrared (SWIR, 1250–2500 nm), the most suitable spectral domain due to the presence of specific physical water absorption features, and near infrared (NIR, 700–1250 nm) [18] or visible (VIS, 350–700 nm) spectral regions [19]–[21]. However, the formulation of a water index can be found in different combinations of bands as well as with different nomenclature for the same formulation. Ji et al. [22] provide a useful review of the existing, and sometimes confusing, nomenclatures of NIR/SWIR water indices and suggest a reference terminology in relation to the portion of the SWIR region being used.

Ouma and Tateishi [23] used Landsat data to perform open water detection and tested several NDSI combinations using a Normalised Difference Water Index (NDWI) nomenclature. They identified the NIR/SWIR normalised difference as the best performing index. The same index was also proposed by Xiao et al. [16] as the Land Surface Water Index (LSWI) and the same formulation is found with other nomenclature: the Normalised Difference Infrared Index (NDII) [24], the Normalised Difference Shortwave-Infrared Index (NDSWIR) [25] and the shortwave infrared water stress index (SIWSI) [26], albeit with reversed band order in the equation.

The capability of NDWI/LSWI to detect water is based on the comparison between the SWIR (1600 nm) and NIR (800 nm) spectral bands (see Table 1) which are sensitive to liquid water thickness – both in open water bodies and vegetation - in the ground area sensed by a radiometer. Even though this NIR/SWIR formulation is useful in detecting water presence, it is theoretically not diagnostic since it cannot separate a flooded soil from plant water content in the presence of a dense vigorous canopy. For this reason, the majority of NIR/SWIR indices, such as the SIWSI and NDSWIR, are proposed for monitoring plant water content/stress or to detect fire-affected areas.

**Table 1:** List of spectral vegetation indices proposed for water detection.

PANEL A							
Spectralrange	SI	Original purpose		Equation	MODIS bands	Reference	Code in thismanuscript
NIR-NIR	Normaliseddifference waterindex	Vegetation liquidwater		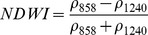	b2, b5	[18]	NDSI_B2B5_
	Normaliseddifference moistureindex	Forest analysis anddetection		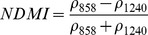	b2, b5	[51]	
	Shortwave infraredwater stress index	Vegetation watercontent		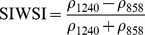	b5, b2	[26]	
NIR-SWIR	Normaliseddifference infraredindex	Vegetation watercontent		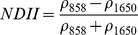	b2, b6	[24]	NDSI_B2B6_
	Normaliseddifferenceshortwave-infraredindex	Identification ofburn scar		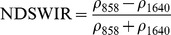	b2, b6	[25]	
	Shortwave infraredwater stress index	Vegetation watercontent		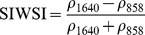	b6, b2	[26]	
	Normaliseddifference waterindex	Change on lakeshorelines		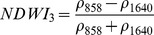	b2, b6	[23]	
	Normalised burnratio	Burn severity		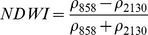	b2, b7	[46]	NDSI_B2B7_
VIS-SWIR	Normaliseddifference waterindex	Open waterdetection		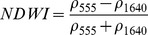	b4, b6	[21]	NDSI_B4B6_
	Modified NDWI	Water detection		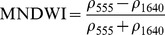	b4, b6	[52]	
	Normaliseddifference pondindex	Detection of smallwater bodies		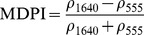	b6, b4	[53]	
	Normaliseddifference waterindex	Water end memberselection		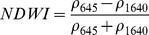	b1, b6	[19]	NDSI_B1B6_
	Normaliseddifference waterindex	Open waterdetection		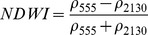	b4, b7	[21]	NDSI_B4B7_
	Normaliseddifference floodindex_2	Flood condition		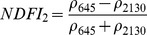	b1, b7	Tested in thisstudy	NDSI_B1B7_
VIS-NIR	Normaliseddifference waterindex	Open waterdetection		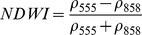	b4, b2	[21], [48]	NDSI_B4B2_
	Normaliseddifference waterindex	Open waterdetection		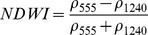	b4, b5	[21]	NDSI_B4B5_
	Normaliseddifference floodindex_1	Flood condition		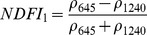	b1, b5	Tested in this study	NDSI_B1B5_
	Normaliseddifferencevegetation index	Water Mapping		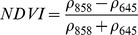	b2, b1	[27]*, [30]**	NDSI_B1B2_
**PANEL B**							
**Spectral combination**		**Original purpose**	**Spectral combination**		**MODIS bands**	**Reference**	**Code in this manuscript**
Combined index		Rice flood mapping	LSWI-EVI^&^		b1, b2, b3, b6	[14], [15] ^&^	B2B6-EVI
Combined index		Rice flood mapping/water bodies andwetland	LSWI-NDVI^&^ OR NDVI-NDWI^$^		b1, b2, b6	[14] ^&^, [16], [29] ^$^	B2B6-NDVI
Enhanced vegetation index		Vegetation monitoring	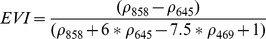		b1, b2, b3	[28]	EVI

Panel A lists the NDSIs tested in this work. For each NDSI is indicated the corresponding mathematical equation and the original reference. Panel B reports equations for combined index B2B6-EVI and B2B6-NDVI calculation.

(*) original formulation for vegetation monitoring;

(**) proposed for water mapping;

(^&^) LSWI (Land Surface Water Index) is equal to the B2B6 MODIS index;

(^$^) NDWI derived from SPOT-VGT data is equal to B2B6 MODIS index.

The influence of vegetation in the LSWI can be removed or at least reduced, thus leaving only the water signal component, by subtracting a vegetation NDSI such as the Normalised Difference Vegetation Index (NDVI) [27] or the Enhanced Vegetation Index (EVI) [28]. Xiao et al. [16] proposed, and applied [14], [17], this concept to detect flooded paddy rice fields using MODIS data if LSWI plus some threshold value is equal to or greater than either NDVI or EVI [14]. A similar approach was adopted by Gond et al. [29] to detect small water areas and humid vegetation in semi-arid areas of Africa. Despite the good performance of this combined index (LSWI-EVI or LSWI-NDVI), it has been noted that flood detection using this approach strongly depends on a locally adapted threshold which has varied from 0.05 [14] to 0.15 (for early-season rice in China) and to 0.21 (for late-season rice in China) in the literature [15]. Calibrating this threshold for different locations, seasons and years is non-trivial, and is one reason to assess flood detection NDSIs with the aim of identifying robust detection methods with fixed thresholds.

Table 1 provides a non-exhaustive summary of the most common indices adopted for water (standing or vegetation) detection. The table indicates the reference and the original purpose of the indices and groups them in relation to the wavelength adopted in the general formulation of an NDSI.
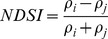
(1)where i and j refer to the different spectral bands used.

In summary NIR-NIR(SWIR) indices are mainly proposed for vegetation water detection while VIS-SWIR combinations are almost all proposed for water detection. Interestingly, the traditional NDVI (VIS-NIR combination) has also been used for water mapping [30].

### 1.2 Objectives

Clearly many water detection NDSIs and associated thresholds are available. We posit that any operational rice detection method which relies on the interpretation of optical time series imagery must have high skill in detecting agronomic flooding with low commission and omission errors. We also posit that this has not received sufficient attention and that there is little guidance on which indices and thresholds are the most robust. To address this, our research tested the performance of a range of NDSIs for detecting water to determine which NDSIs were most suitable for agronomic flood detection across various rice agro-ecosystems. Hence, in this study we make an intercomparison of NDSIs from table 1 and additionally we compare the NDSIs against the combined LSWI-EVI which we refer to as the B2B6-EVI index.

The objective of this research was to identify and assess robust flood detection NDSIs that met three criteria. The indices must (i) provide diagnostic information on the presence of a thick water layer, (ii) have minimal confusion with plant/soil water content and (iii) be able to separate the surfaces (water, soil, vegetation) for automatic detection purposes.

To reach these objectives, we performed a number of tests and experiments using both field and satellite data. Unlike previous studies such as Ji et al. [21], we:

- compared a range of NDSIs available in the literature, including new combinations of VIS(GREEN/RED)-SWIR spectral bands as well as the combined B2B6-EVI index;
- used real spectroradiometric measurements acquired by field and satellite data (rather than simulated data from simple linear combinations of spectral library data) to evaluate mixed pixel effects on water indices;
- used MODIS data in conjunction with Landsat data at four different sites to evaluate the NDSI performance in real-world conditions where land cover mixture among vegetation, bare soil and water can alter the signal; and
- analysed the performance of the NDSIs on MODIS time series in four study areas.

This is the first time that published NDSIs and related thresholds for water detection have been compared, calibrated, validated and ranked across different environments on real satellite MODIS data. This analysis is necessary as a preliminary study for a remote sensing-based operational rice detection and monitoring system starting from the detection of agronomic flooding in a complex and mixed land cover environment. In all stages, of the analysis we compare the NDSIs to the performance of the combined B2B6-EVI index. A further comparison against the combined B2B6-NDVI index is shown in File S1.

## Materials and Methods

Remote sensing analysis of different crop growth and bare soil conditions (dry, moist, saturated or flooded) was conducted by exploiting both field and satellite data. Field measurements provided accurate data under the controlled conditions necessary to test hypotheses on NDSI performance in different physical conditions. Satellite data were used to assess the applicability of the field data findings in real-world conditions by (i) testing the accuracy of NDSI water detection in pure water pixels, (ii) analysing the impact of different mixed pixel conditions on NDSI performance and (iii) evaluating NDSI behaviour on multi-years MODIS time series. The satellite data were acquired in different locations and different rice cropping systems to permit an evaluation of the NDSIs under different environments and management conditions.

### 2.1 Field Data

Field spectral data were acquired with Field Spec® Full Range spectro-radiometer (Analytical Spectral Device Inc., U.S., 350–2500 spectral range at 1 nm spectral resolution) in rice fields in an agricultural district of northern Italy. This district provides most (>95%) Italian rice production and about 60% of the total European rice production [31]. Two different data sets were exploited: (i) an archive of spectral measurements acquired from two agronomic experiments, carried out on private land, in 2004 and 2006 at a site located south of Milan, Opera (45°23′ N, 9°11′ E), and (ii) a data set from 2011 under real farm conditions in Pavia province in several fields (site 1: 45°10′N, 9°6′E; site 2: 45°10′N, 9°7′E; and site 3: 45°8′N, 9°9′E) characterised by different soil conditions (dry soil, wet soil and flooding) before sowing and/or plant emergence. For indirect field data acquisition, like spectral measurements, in these locations a specific permission was not required. Five spectra were collected along a transect in each plot/site preceded and followed by a measurement on a reference panel (Spectralon®). Data were collected in nadir direction, about 1 m above the canopy, between 12∶00 h and 13∶00 h under stable solar conditions. The vegetation/soil reflectance was derived using the following formula:
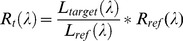
(2)where *R_t_*(λ) is the target (vegetation, soil or water) reflectance, *L_target_* (λ) is the radiance measured above vegetation/soil/water, *L_ref_* (λ) is the radiance of the reference panel and *R_ref_* (λ) is the specific calibration reflectance coefficient of the reference panel.

Table 2 reports a synthesis of the field data, including GPS coordinates of each location, and auxiliary measurements that were conducted simultaneously with the spectral measurements. Further details on the experimental design of the 2004 and 2006 field data sets as well as the measurement protocols are provided in [32], [33].

**Table 2:** Field data characteristics and cardinality of available spectra for each data set (#).

Data set	Year	Location	Lat. (N)	Lon. (E)	Samplingarea	Field conditionand measure	Additional measure
Experimental data set	2004*	Opera	45°23′	9°11′	1 field,40 plots$	Rice in flooded field (# 320)	LAI/fAPAR, Nitrogen, SPAD, picture
	2006**	Opera	45°23′	9°11′	1 field	Dry soil (# 4)	Water level, picture
					2 field	Wet soil (# 10)	Water level, picture
					3 field	Flood soil& ^,^ £ (# 24)	Water level, picture
					1 field	Rice& (# 6)	Water level, picture
Farm conditions data set	2011***	Carbonara Ticino	45°10′	9°6′	1 field§	Dry soil (# 18)	Water level, picture
		San MartinoSiccomario	45°10′	9°7′	1 field§	Dry soil (# 16)	Water level, picture
					2 field§	Flood soil (5–10 cm) (# 37)	Water level, picture
		Travacò Siccomario	45°8′	9°9′	1 field§	Flood soil (15 cm) (# 18)	Water level, picture

*eight weekly measurements from 17 June to 16 August;

**28 June;

***19 May.

$ 2 varieties, 4 replicates, 5 treatments (40 plots);

& Different water height (3–10 cm),

£ Presence of algae (# 16).

§ 1 transect per field, about 15 spectra for each transect.

### 2.2 Satellite Data and Study Sites

Four Landsat images, acquired over rice-producing regions in Asia and Europe, were processed to provide a spatial representation of the physical status of rice cultivated area at high resolution (30 m). The four images represent well-known rice areas: Piemonte-Lombardia regions (Italy) – the largest rice-growing areas in Europe, West Godavari (India) – a highly productive district in Andhra Pradesh, the Mekong delta (Vietnam) – the major rice-producing region of the country, and Siem Reap (Cambodia) – where rice is cultivated extensively. Each area has distinct geography, environment and crop management practices that represent most of the variation in rice systems.

The Italian site (ITA) is situated at the confluence of Sesia, Ticino and Po rivers where rice is cultivated once per year in irrigated and highly managed cropping systems. The crop cycle lasts for 130–150 days from the second half of April/early May (seeding period) to the end of September (harvesting) [34]. Flooding starts in April and finishes at the end of August. Fields are either flooded a few days before seeding for wet seeding or flooded three weeks after seeding for dry seeding.

The Indian site (IND) is situated in the southern state of Andhra Pradesh, where irrigated rice is mainly grown in the deltaic tracts of the Godavari river [35]. Up to three crops per year are possible and two of these may be cultivated with rice [36]. Rice sowing occurs in May–June for the *Kharif* or monsoon season, and December–January for the *Rabi* or winter season.

The other two Asian sites, Cambodia (KHM) and Vietnam (VNM), are located in the Lower Mekong river basin. The monsoon rain from May to October leads to annual flooding along the Mekong river from August to December [12]. The KHM site includes Tonle Sap Lake, where rice cultivation starts in January–February after the long monsoon flood recession [37]. The Mekong Delta site (VNM) can have up to three irrigated rice crops per year with three flooding periods, November–December (winter–spring season), April–June (summer–autumn season) and August (autumn–winter season) [38].

The US Geological Service (USGS) archive – Global Visualization Viewer (GloVis; http://glovis.usgs.gov/) – was used to select appropriate Landsat images for these sites. Acquisition dates were selected according to the above rice season information to capture flooded rice fields at the beginning of the crop season (Table 3). We selected Landsat images from April 2003 in Italy (path-row 194-28/29), January 2002 in India (path-row 142-48) and November 2000 in both Vietnam and Cambodia (path-row 125-53 and 127-51, respectively). We selected subsamples within each Landsat footprint to capture rice areas and surrounding areas with other crops, natural vegetation, bare/wet soil conditions and water bodies. These subsamples provided good representative areas for testing the skill of the NDSIs for rice flood detection.

**Table 3:** Study areas: satellite data available and selected location used to study the temporal series of NDSIs.

Land use		MODIS Terra		Landsat 7 ETM+			Location for temporal series			
Sites	*# rice seasons*	*Tile*	*date* *	*path-row*	*date*	*Analysed area [km]*	*Lon. (E)*	*Lat. (N)*	*MODIS Row*	*MODIS Column*
ITA	Single	h18v04	25/04/03	194-28/29	24/04/03	80×63	8.26°	45.24°	1143	1396
VNM	Triple	h28v07	09/11/00	125-53	07/11/00	187×85	105.54°	10.14°	2367	934
IND	Double	h25v07	01/01/02	142-48	03/01/02	180×87	16.70°	81.84°	792	2014
KHM	Single	h28v07	01/11/00	127-51	05/11/00	138×60	103.71°	13.53°	1553	200

*Reference composite date.

The MODIS TERRA MOD09A1 500-m product, validated version collection V005, was selected to perform NDSI analysis. The MOD09A1 product provides an 8-day composite in seven surface reflectance spectral bands in the RED (630–690 nm; b1), NIR (780–900 nm; b2), BLUE (450–520 nm, b3), GREEN (530–610 nm, b4), NIR (1230–1250 nm; b5), SWIR (1550–1750 nm, b6) and SWIR (2090–2350 nm, b7) wavelengths. Science Data Sets provided for this product include a pixel-specific quality assessment and the day of the year (DOY) with solar view and zenith angles of the acquisition selected in the composite. All data are geocoded in the Sinusoidal projection. Composite data for the tiles that cover southern Europe (h18v04), eastern India (h28v07) and South-East Asia (h25v07) were downloaded for the four-year period (2000–2003) in order to have the same temporal and spatial extent as the Landsat images. NDSIs were calculated for each MODIS scene.

Finally, within each study site we selected locations – based on the Landsat imagery – where rice cultivation was clearly visible and we obtained four years (2000–2003) of MOD09A1 time series data from the University of Oklahoma web service (www.eomf.ou.edu/visualization/gmap/).

**Figure 1:**
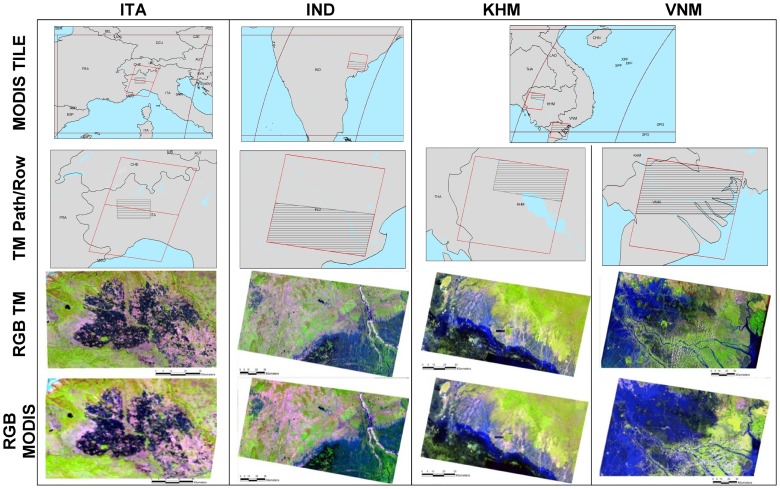
Study areas and satellite data analysed for the four case studies (ITA, IND KHM, VNM). Black and red boxes indicate, respectively, the MODIS tile (H18v04,H25v07 and H27v07) and Landsat (194-28/29, 125-53, 142-48 and 127-51) extent. Black lines indicate the analysed area for which a Landsat – RGB 543- and MODIS-RBG 621– colour composite is provided.

Figure 1 reports a synthetic view of the selected study areas and satellite data. Table 3 shows information on the MODIS and Landsat images that were analysed, indicating the geographic location (countries and coordinates) as well as image reference (tiles and pixel coordinates) and the number of rice seasons per year.

### 2.3 Pre-processing and Analysis of Field Data

In order to simulate the response of the MODIS sensor, the spectral reflectance collected on the ground was resampled with the specific MODIS spectral response functions. The band-equivalent MODIS reflectance (R_MODISi_) was derived by integration over each band’s spectral response function using IDL code (*EXELIS, Visual Information Solution*). MODIS reflectances (R_MODISi_) simulated from field data were combined to calculate the NDSIs (Table 1). NDSIs are labelled using their first and second wavelengths λ_1_λ_2_, i.e. b1.b2 when MODIS Band1 (RED) and MODIS Band2 (NIR) are used, and grouped by spectral range as VIS/NIR (NDSI_b4.b2_, NDSI_b1.b2,_ NDSI_b4.b5,_ NDSI_b1.b5_), VIS/SWIR (NDSI_b4.b6_, NDSI_b4.b7_, NDSI_b1.b6_,NDSI_b1.b7_), NIR/NIR (NDSI_b2.b5_) and NIR/SWIR (NDSI_b2.b6_, NDSI_b2.b7_).

One-way analysis of variance (ANOVA) followed by the post-hoc Tukey HSD (honestly significant difference) multiple range test were used to compare NDSI responses to different targets (flooded field, wet soil, dry soil and vegetation) in order to highlight which NDSI was more sensitive and theoretically more robust in detecting and differentiating between targets. The vegetation class was divided into two sub-classes for leaf area index (LAI): less than or equal to 2.0 representing a sparse canopy (i.e. a young rice crop or shortly after transplanting) and greater than 2.0 representing full canopy cover. Statistical analysis was performed using the R software environment (R version 2.15.0, packages agricolae and stat; www.r-project.org/). Finally, in order to assess the skill of each index in separating water surfaces from other surfaces, we computed a measure of separability, *S*:
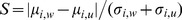
(3)where µ_i,w_ and σ_i,w_ are, respectively, the mean and standard deviation of fully flooded conditions and µ _i,u_ and σ_i,u_ are, respectively, the mean and standard deviation of other surfaces: pure soil, full cover vegetation or mixed conditions. The *S* statistic assesses the degree of discrimination between two groups. A low value of *S* (<1) denotes that the distribution of the classes overlap significantly and the ability to separate (or discriminate) the two groups is poor. A high value of *S* (>>1) denotes that the means are well separated and that the two classes are relatively easy to discriminate. The numerator of the *S* statistic describes the difference in mean values between the two considered classes, while the denominator provides a degree of noise/dispersion of the classes. Classes with larger σ’s have wider distribution, lower S values and thus will be more likely to overlap. Therefore, the *S* statistic can be effectively used to compare the ability of different NDSIs to discriminate between water and other classes.

### 2.4 NDSI Satellite Data Analysis

#### 2.4.1 Threshold definition and evaluation on pure pixel condition

The subsample of each Landsat 7 ETM+ image, centered on a rice-growing area with evident flood condition, was selected (Figure 1 and Table 3) and classified by unsupervised ISODATA algorithm (*EXELIS, Visual Information Solution*) without performing any atmospheric correction [39]. Subsampling at the KHM site was particularly necessary to exclude strongly cloud contaminated areas while at the VNM site we analysed only the Landsat image that overlapped the MODIS tile. We characterised the four sites into three land cover (LC) classes describing the surface physical condition at the time of the satellite overpass: water (mainly representing the typical condition of agronomic flooding in rice cultivation but also river and water bodies), vegetation (natural system and established crops) and bare soil (dry or wet). We also identified a no data class corresponding to cloud cover and shadow. These high-resolution land cover (LC) maps were used to characterise the composition of each MODIS pixel and to interpret the changes in NDSI values in relation to mixture components. To perform this analysis, a random selection of more than 3500 MODIS pixels was extracted for each site, thus creating a database that recorded for each pixel the percentage of different land cover classes as provided by LC maps as well as the MODIS band response and NDSI values.

From this database, we selected 4000 pure MODIS pixels – distributed equally across each site –2000 of water and 2000 of no water (1000 of soil and 1000 of vegetation). In order to define thresholds for surface water detection with different NDSIs, this 4000 pixel sample was randomly split into two samples equally distributed across each site and across LC classes: the first group was used for calibration of NDSI thresholds and the other for validation of the NDSI classification performance.

We used a recursive partitioning technique implemented in R (rpart package) to identify the best performing threshold for each analysed NDSI. The partitioning is based on minimal error fitting on the calibration data set and it identifies the NDSI value that best discriminate pure flooded pixels from pure vegetated or pure soil pixels.

Accuracy of the classification provided by the identified thresholds was assessed by the metrics computed from the error or confusion matrix using the validation/testing set: Overall Accuracy (OA), Commission Error (CE) and Omission Error (OE) were calculated to identify the best performing NDSI. Accuracy was also expressed in terms of the kappa coefficient of agreement and a Z-test was used to evaluate the significance of differences in classification outputs [40].

#### 2.4.2 Mixture effect analysis

To analyse the effect of different subpixel water percentages on NDSI values, we extracted a stratified random sample of 625 MODIS pixels from each of the four test sites. The 625 pixels were equally distributed across five classes of MODIS subpixel water percentage (100-80%, 80-60%, 60-40%, 40-20%, 20-0%); consequently, a data set of 2500 (125×5×4) pixels was obtained.

This selection was used in a second-order polynomial regression between the NDSI values and MODIS subpixel water percentage. Noise equivalent (NE) (Eq. 4) was then computed to measure the sensitivity of each NDSI to changes in relation to water percentage:
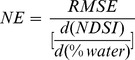
(4)where RMSE is the root mean square error of the water percentage-NDSI regressive model and d(NDSI)/d(%water) is the first derivative of the NDSI with respect to water percentage. NE was chosen since it permits a direct comparison of indices with different scales and dynamic ranges [41]–[43]. NE provides a measure of how well the NDSI responds to water percentage across its entire range of variation. A low NE value means that an NDSI is sensitive to water presence.

#### 2.4.3 Mapping performance in mixture conditions

Thematic cartography produced by the automatic classification of low-/medium-resolution satellite data can be affected by low-resolution bias when compared to high-resolution reference data [44]. Low-resolution bias is the inaccuracy introduced by the difference in spatial resolution between high- and low-resolution data and is not related to the performance of a classification algorithm. The bias, linked to the characteristics of the features on the ground and a function of shape, size and fragmentation of the target under analysis, can be computed for each test site using a Pareto boundary analysis [44]. This approach assesses the potential maximum accuracy that a classification method can achieve in a specific experimental condition as a function of the actual fragmentation of the environment. Thus it can be used to rank the performance of the NDSIs.

For each site we derived moderate resolution surface water maps from each NDSI using the identified thresholds (§2.4.1) to evaluate the performance of the NDSIs in real conditions where mixed pixel problems can affect classification. These moderate resolution NDSI water maps were compared with the high resolution LC map derived from Landsat for each site and their performance was quantified with respect to the Pareto boundary in the Commission Error (CE) – Omission Error (OE) space.

## Results and Discussion

**Figure 2:**
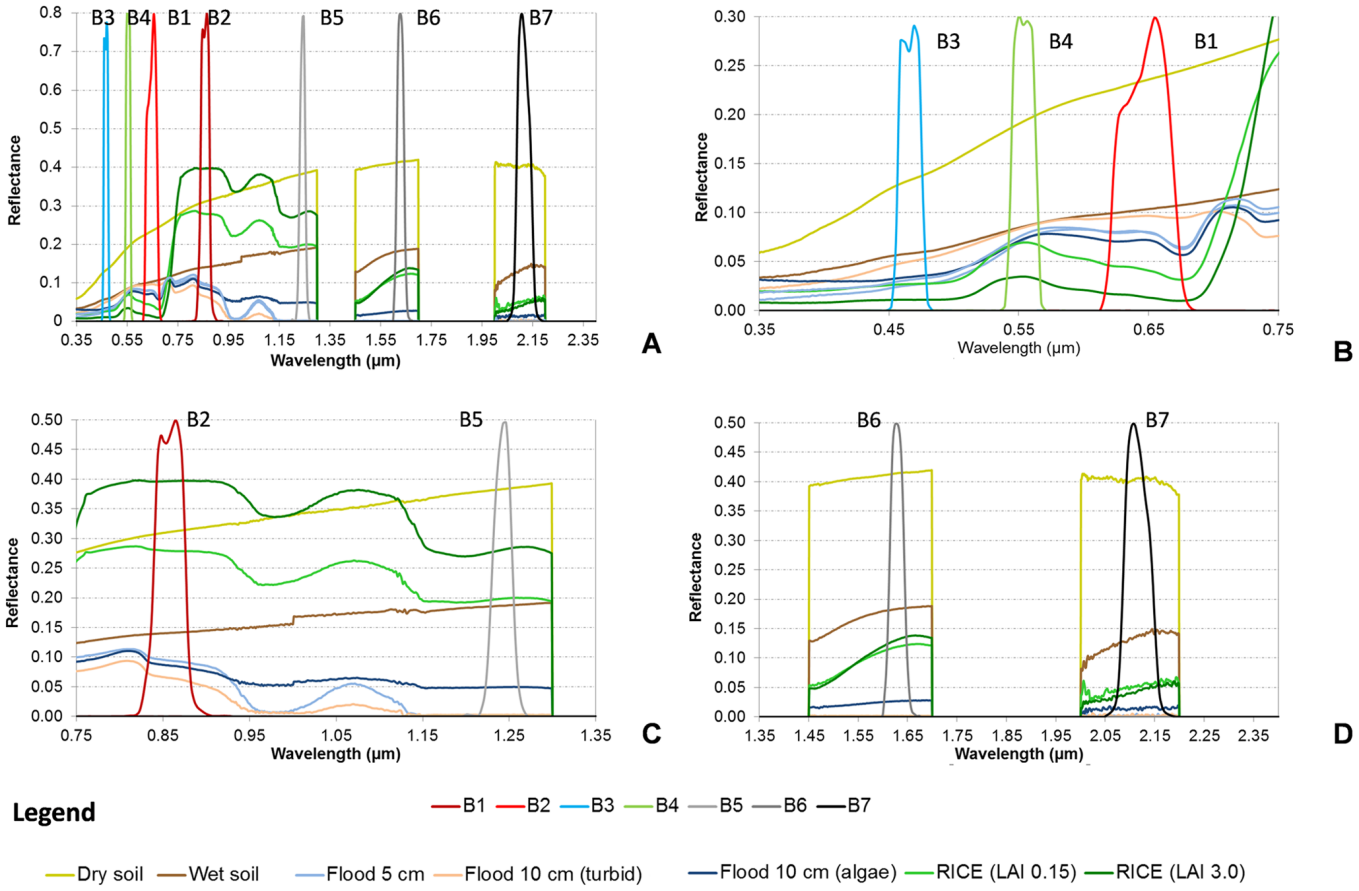
Comparison of different surface responses in different spectral ranges: full range 350–2500 (a), visible [0.35–0.75] (b), near infrared [0.75–1.35] (b) and shortwave infrared [1.4–2.5] (b). The position of MODIS spectral response function of bands 1–7 is overlayed on the field data.

Figure 2 shows the field spectral data for different surfaces analysed together with the position of the seven MODIS spectral response functions in the range 350–2500 nm. Figure 2a shows the entire spectral range for which data were acquired; Figure 2b, 2c and 2d provide details for the VIS, NIR and SWIR regions, respectively.

In the visible (VIS) range (MODIS bands B1, B3 and B4), the wet soil and flooded field conditions have similar spectral responses, with a slight increase from short to long visible wavelengths. An increase in water presence, from saturated soil to a 5 to 10 cm deep water layer, reduces the reflectance, mainly in the RED region, but with a lesser effect when compared to the change observed between dry and wet soil. Dry soils show a typical increase, with the highest reflectance for RED (up to 25%). On the contrary, vegetation behaviour presents the lowest reflectance in the photosynthetically active radiation region (PAR, 350–700 nm), confirming the typical absorption features which correspond to the BLUE and RED bands due to chlorophyll action. It is interesting to note that, when water, between 5 and 10 cm deep, is present on soil, the main changes in the VIS region are due to the presence of algae or suspended matter. Algae in flooded soil show an absorption feature in RED at 670 nm, whereas turbid water behaves similarly to saturated soil and exhibits high reflectance. The B1 RED band shows the strongest differences between the three land conditions of dry soil, flood/wet soil and vegetation (Figure 2b).

In the near infrared (NIR) range (B2 and B5), the surfaces have a different albedo behaviour: B2 shows high values for dense and healthy vegetation conditions, followed by dry soil, low dense vegetation, wet soil and flooded. Band B5 shows a strong sensitivity to water content and drops are visible in vegetation and flooded soil. The presence of algae increases the reflectance in this spectral range; a flood condition shows values close to zero (Figure 2c). Indeed, for wavelengths greater than 1100 nm, the presence of a water layer produces the largest differences between the spectra.

In the shortwave infrared (SWIR) range (B6 and B7), water absorption features dominate the spectral response. The absorption coefficient increases with wavelength in an exponential way with specific high peaks at 1400 and 1925 nm [45]. Flooded areas present a signal close to zero even when algae or sediments are present. The vegetation spectral response also decreases markedly, reaching an absolute value of 5%, showing a reflectance two-thirds lower than wet soil (Figure 2d).

### 3.1 NDSI Analysis from Field Data

**Figure 3:**
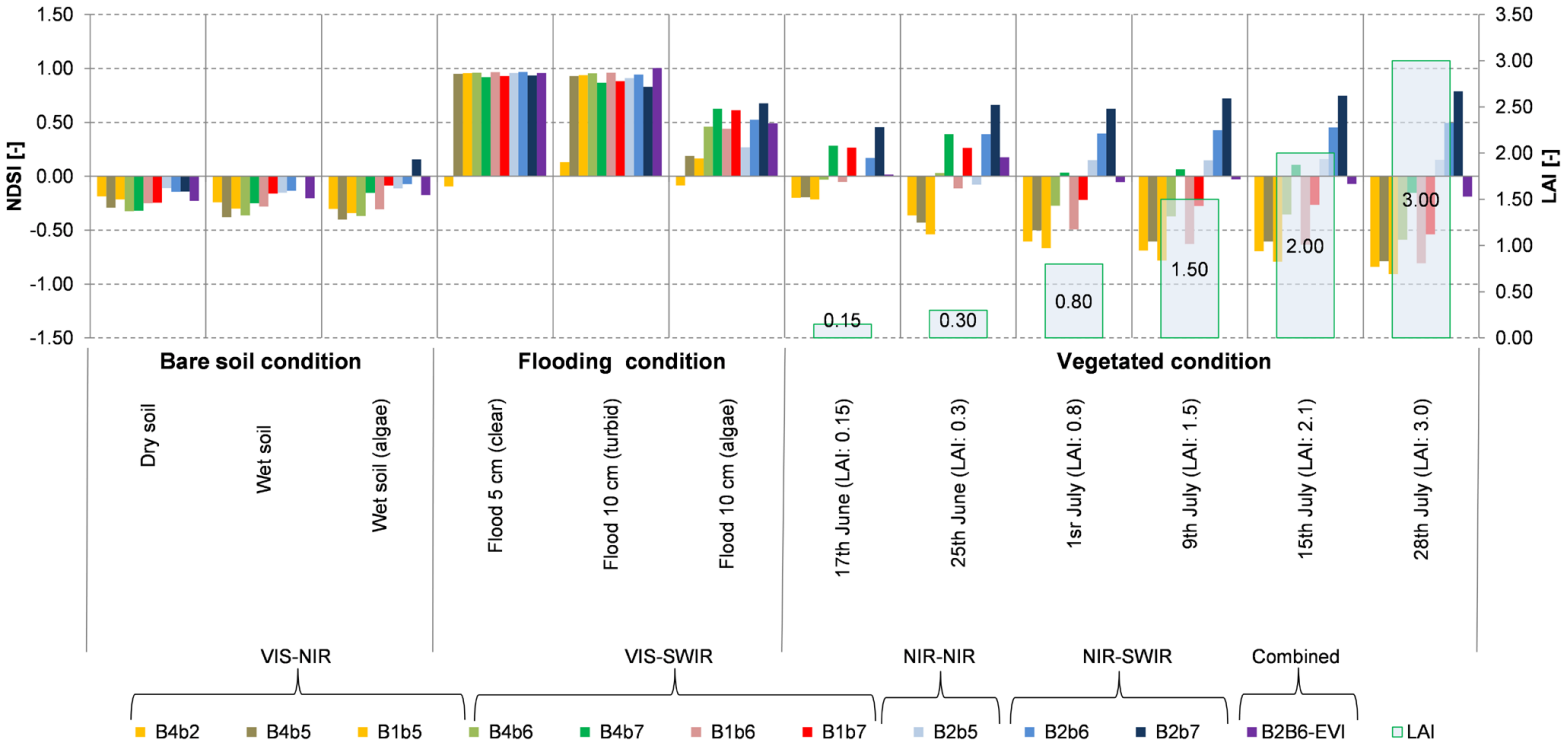
Behaviour of spectral indices (NDSIs) in different paddy rice conditions from bare, dry soil to dense vegetation.

Figure 3 shows the values of the tested NDSIs calculated on the R_MODISi_ that was simulated from the field spectra acquired in 2004 and 2005 in the experimental field. The data – from left to right – represent a plausible temporal development of paddy conditions during a crop season, ranging from bare soil to flooding and then through the vegetative stages from germination to tillering prior to flowering. NDSIs – grouped by wavelength region combinations – are represented on the primary y axis in yellow, green/red and blue tones for VIS/NIR, VIS/SWIR, NIR/NIR and NIR/SWIR, respectively. The combined B2B6-EVI index is shown in purple. The secondary y axis shows field LAI in green to provide information on the status of the rice crop in terms of biomass and development.

Table 4 reports the results of the ANOVA and post-hoc Tukey test to compare NDSI responses among different targets. Classes that are significantly different (*p*<0.01) from each other in terms of NDSI values are indicated with different letters. Table 5 reports the values of the separability metric (*S*) computed between flooded condition (W) and each of wet soil (WS), dry soil (DS), sparse canopy cover (V1) and full canopy cover vegetation (V2) for each NDSI and the combined B2B6-EVI index. A global score for separability is provided in terms of both average separability value (S_AVG_) and ranking position (Rank_AVG_), where rank is from best (1) to worst (12) separability performance between the different surfaces.

**Table 4:** Results of ANOVA between flood condition (W) and other classes: wet soil (WS), dry soil (DS), sparse canopy (V1) and full canopy cover vegetation (V2) and post-hoc Tukey test.

		VIS/NIR												VIS/SWIR														NIR/NIR			NIR/SWIR							COMPOSED		
		B4B2			B1B2			B4B5			B1B5			B4B6			B4B7				B1B6			B1B7				B2B5			B2B6				B2B7			B2B6-EVI		
Class	r	µ	σ	G	µ	σ	G	µ	σ	G	µ	σ	G	µ	σ	G		µ	σ	G	µ	σ	G		µ	σ	G	µ	σ	G		µ	σ	G	µ	σ	G	µ	σ	G
W	79	−0.01	0.02	**a**	0.04	0.02	**a**	0.69	0.04	**a**	0.75	0.04	**a**	0.78	0.03	**a**		0.74	0.02	**a**	0.75	0.04	**a**		0.78	0.03	**a**	0.71	0.030	**a**		0.80	0.030	**a**	0.75	0.020	**a**	0.85	0.05	**a**
WS	10	−0.35	0.05	**bc**	−0.33	0.07	**b**	−0.28	0.13	**b**	−0.04	0.14	**b**	−0.21	0.13	**b**		−0.03	0.11	**bc**	−0.04	0.10	**b**		−0.21	0.10	**b**	0.05	0.100	**b**		0.13	0.100	**cd**	0.30	0.100	**b**	−0.56	0.15	**bc**
DS	38	−0.20	0.01	**b**	−0.12	0.007	**ab**	−0.31	0.02	**b**	−0.29	0.01	**b**	−0.36	0.02	**b**		−0.37	0.02	**c**	−0.29	0.02	**b**		−0.36	0.01	**bc**	−0.12	0.006	**b**		−0.17	0.009	**d**	−0.18	0.009	**c**	−0.42	0.022	**b**
V1	35	−0.49	0.03	**c**	−0.61	0.03	**c**	−0.40	0.03	**bc**	−0.07	0.04	**bc**	−0.20	0.03	**b**		0.14	0.03	**b**	−0.07	0.04	**b**		−0.20	0.04	**b**	0.11	0.030	**b**		0.33	0.020	**bc**	0.58	0.030	**ab**	−1.8	0.025	**d**
V2	35	−0.76	0.01	**d**	−0.90	0.01	**d**	−0.70	0.02	**c**	−0.48	0.01	**c**	−0.48	0.02	**b**		−0.06	0.02	**bc**	−0.48	0.01	**c**		−0.48	0.02	**c**	0.14	0.009	**b**		0.46	0.010	**b**	0.74	0.009	**a**	−1.1	0.085	**c**

Letters indicate statistically different groups (α = 0.01).

**Table 5:** Separability (S) between flood condition and other classes: wet soil (WS), dry soil (DS), sparse canopy (V1) and full canopy cover vegetation (V2).

	VIS/NIR								VIS/SWIR								NIR/NIR		NIR/SWIR				COMBINED	
	B4B2		B1B2		B4B5		B1B5		B4B6		B4B7		B1B6		B1B7		B2B5		B2B6		B2B7		B2B6-EVI	
Class	S	Rank	S	Rank	S	Rank	S	Rank	S	Rank	S	Rank	S	Rank	S	Rank	S	Rank	S	Rank	S	Rank	S	Rank
WS	1.20	8	0.93	11	1.27	6	1.22	7	**1.40**	**1**	1.30	5	1.38	3	1.39	2	1.10	10	1.14	9	0.82	12	1.35	4
DS	0.96	11	0.77	12	2.27	9	2.14	10	2.79	6	3.38	2	2.64	7	3.22	4	2.64	8	3.25	3	**3.74**	**1**	2.89	5
V1	1.63	8	1.80	6	1.99	4	**2.10**	**1**	2.01	3	1.52	9	2.08	2	1.67	7	1.39	10	1.26	11	0.47	12	1.92	5
V2	3.46	4	**4.61**	**1**	3.19	6	3.81	3	3.15	7	2.27	9	4.01	2	3.44	5	1.76	10	1.12	11	0.02	12	2.76	8
S_AVG_	1.81		2.03		2.18		2.32		2.34		2.12		**2.53**		2.43		1.72		1.69		1.26		2.23	
RANK_AVG_	7.75		7.50		6.25		5.25		4.25		6.25		**3.5**		4.5		9.5		8.5		9.25		5.5	

SAVG refers to the average separability value of each index for water with respect to all the other classes. Rank refers to the relative position (1 = best and 12 = worst) of each index separability with respect to the others and RankAVG the mean value.

It is interesting to note that, despite some specific differences, the NDSIs belonging to the same spectral category behave in a similar way. NDSIs in VIS-SWIR (B4B6, B4B7, B1B6 and B1B7) have significantly different values for water with respect to the other classes (see groups in ANOVA analysis, Table 4) and hence high *S* values (S_AVG_: 2.34, 2.12, 2.53 and 2.43; Table 5) and first positions in separability ranking (RANK_AVG_: 4.25, 6.25, 3.5 and 4.5; Table 5). These indices are clearly diagnostic of water surfaces, having positive values only for flood conditions and negative values for soil (both wet and dry) and vegetation cover. The only exception is B4B7 that has positive values for sparse vegetation but is still significantly different from water conditions.

Also, VIS/NIR NDSIs that use band 5 (B1B5 and B4B5) show a performance similar to that of the previous category in terms of average separability (2.32 and 2.18) and average ranking position (5.25, 6.25). However, we observe that B4B5, that was proposed by [21] based on their analysis of spectral library data is ranked sixth both for separability score and average rank.

The post-hoc test shows that both B2B7 and B1B2 cannot separate water from all the other classes. B2B7 fails to distinguish water from vegetation and BIB2 fails to distinguish water from dry soil, even if they show the highest separability performance with respect to dry soil (B2B7) and dense vegetation (B1B2).

B2B5 is able to separate water from other surfaces and negative values are reported only for dry soil conditions. Gao [18] proposed this index to monitor liquid water in vegetation and it is based on water absorption features at 1240 nm. The results are therefore in agreement with the theoretical assumption: higher values must be interpreted as higher water presence (W>>V2>V1>WS; Table 4). The B2B6 results can be interpreted in a similar way; however, the use of a SWIR band with longer wavelength (1600 nm), where water absorption is stronger, determines that vegetation values are no longer significantly different from those of wet soil. This index also has negative values for dry soil and can easily separate bare soil conditions from flooding (*S* = 3.25; rank = 3).

Finally, B2B7, similar to B2B5 and B2B6, easily separates water from dry soil (*S* = 3.74; rank = 1) but is not able to differentiate classes that present medium/high wet conditions, and dense canopy cover has values that are similar to those of water (*S* = 0.02; rank = 12). The B2B7 index was originally proposed to evaluate burn severity on vegetated areas as a consequence of water loss [46]. It is important to highlight that these last three indices were developed and adopted to monitor water content, primarily in vegetation, and are therefore sensitive and strongly correlated to the total amount of water that can be remotely sensed.

The combined B2B6-EVI index is a good indicator of water condition. ANOVA demonstrates that the flood condition is statistically different from other classes and the separability analysis shows an average value that is comparable to the VIS/SWIR categories though it is placed fifth in the ranking.

### 3.2 NDSI Analysis from Satellite Data

The high-resolution maps, derived from Landsat (see §3.4), show agricultural landscapes dominated by the agronomic flooding of rice fields but with a certain degree of heterogeneity as a consequence of different timing in field management activities (i.e., flooded or bare soil conditions) and the presence of other crops and natural vegetation patches. It is this heterogeneity of land cover and land use that permits an analysis of MODIS NDSI performance in real-world conditions. We first report the results of the pure MODIS pixel separability analysis with threshold definition and then the mixed MODIS pixel NDSI sensitivity analysis.

#### 3.2.1 Separability analysis on MODIS pixels

Table 6 shows the results of the separability analysis conducted on 2000 pure MODIS pixels to determine which NDSIs best discriminate pure water pixels from other LC classes. The separability and ranking are given for each test site as well as average values across sites. The NDSIs of the VIS/SWIR family (B4B6, B4B7, B1B6, and B1B7) outperform the other NDSIs consistently across sites. In particular, B1B7 and B4B6 are the best across all sites (Rank_AVG_: 3.255) but these NDSIs have the highest ranking position only for KHM (B1B7), whilst B4B6, B4B7 and B1B6 are ranked second in ITA, IND and KHM/VNM respectively. Only the B1B5 NDSI, from the VIS/NIR family, outperforms the VIS/SWIR category at the VNM site (S: 3.9, Rank: 1).

**Table 6:** Separability (S) score between pure water MODIS pixels and other LCs (soil and vegetation) for the different NDSIs and combined index at all test sites.

	VIS/NIR								VIS/SWIR								NIR/NIR		NIR/SWIR				Combined	
	B4B2		B1B2		B4B5		B1B5		B4B6		B4B7		B1B6		B1B7		B2B5		B2B6		B2B7		B2B6-EVI	
Site	S	Rank	S	Rank	S	Rank	S	Rank	S	Rank	S	Rank	S	Rank	S	Rank	S	Rank	S	Rank	S	Rank	S	Rank
IND	2.0	11	1.3	12	3.3	6	2.6	10	4.2	3	4.5	2	3.3	5	4.0	4	3.2	7	3.1	8	3.0	9	**4.7**	**1**
ITA	1.6	10	1.3	12	2.6	7	2.1	8	3.5	.2	3.4	4	2.7	5	3.4	3	2.7	6	1.8	9	1.5	11	**4.8**	**1**
KHM	1.5	10	1.6	8	1.8	7	2.0	6	2.0	5	2.2	3	2.2	2	**2.6**	**1**	1.5	9	0.5	12	−0.2	11	2.2	4
VNM	2.5	8	2.8	7	3.6	3	**3.9**	**1**	3.5	4	3.3	6	3.8	2	3.5	5	1.8	10	1.4	11	1.2	12	2.1	9
S_AVG_	1.90		1.80		2.80		2.70		3.30		3.30		3.00		**3.40**		2.30		1.70		1.40		**3.40**	
RANK_AVG_	9.75		9.75		5.75		6.25		3.5		3.75		3.5		**3.25**		10		12		10.75		3.75	

SAVG refers to the average separability value of each index for water with respect to all the other classes at the four sites. Rank refers to the relative position (1 = best and 12 = worst) of each index separability with respect to the others and RankAVG the mean value.

These results agree with the analysis of field data: the VIS/SWIR family performs better than other NDSIs. Among them, the average separability value indicates a slightly better performance for B1B7 (S_AVG_: 3.4) and B4B7 (S_AVG_: 3.3), both based on longer SWIR wavelength, followed by B4B6 (S_AVG_: 3.3) and B1B6 (S_AVG_: 3.0).

The analysis of the combined index B2B6-EVI on real MODIS data confirmed the field data results. This index performs well with a S_avg_ value equivalent to B1B7. However, this value is a consequence of B2B6-EVI being the best ranked index for IND and ITA, whilst in KHM and in particular in VNM the index is not highly ranked. This variability in ranking across sites, which is higher than the ranking variability in any of the VIS/SWIR indices, suggests that the combined B2B6-EVI index is sensitive to local conditions and supports the previous statements on the need for locally adaptive thresholds for this index.

#### 3.2.2 Threshold calibration and validation

According to the separability analysis, NDSIs of the VIS/SWIR family have greater skill in distinguishing water from other surfaces. However, a further comparison was made using a calibration approach to define the threshold on the basis of a training data set and validating the results on a separate/independent test data set.

**Figure 4:**
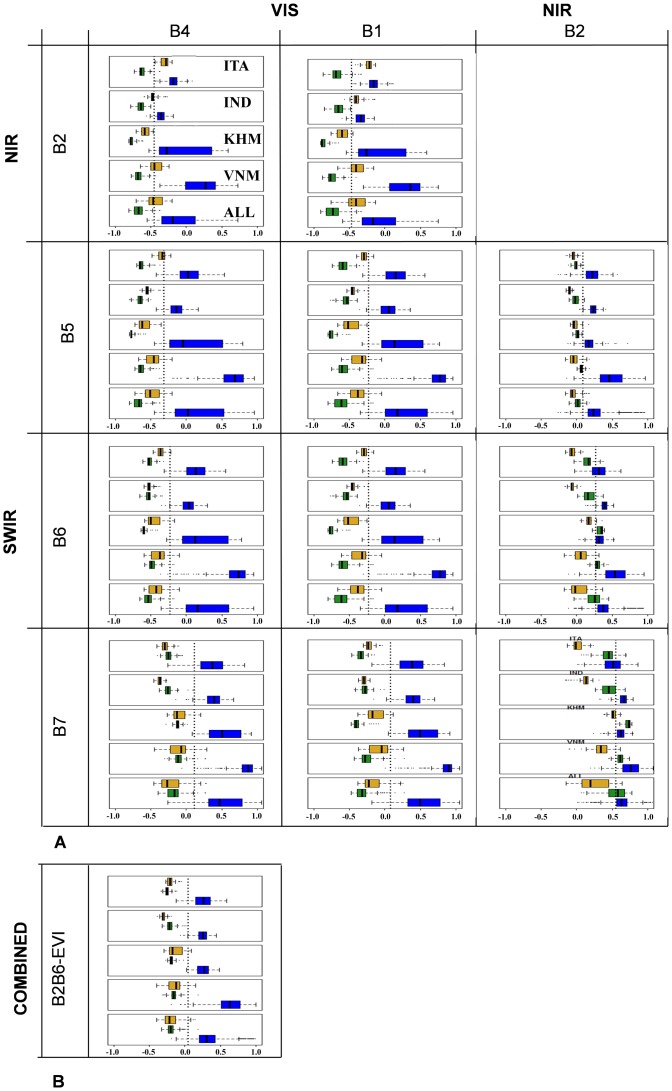
Boxplots of analysed indices for pure water pixels (blue), pure soil pixels (orange) and pure vegetation pixels (green). Every boxplot has the same cardinality. The vertical dotted lines show the calculated threshold. Panel (a) shows the NDSIs and Panel (b) the combined B2B6-EVI index.

Figure 4 shows the boxplots of the calibration data set results in a matrix layout. The rows and columns of the matrix represent the possible NDSI band combinations, while each matrix element shows the boxplots of the respective NDSI values for water, soil and vegetation pixels for each site (500 training samples each for ITA, IND, KHM and VNM) and ‘globally’ across all sites (2000 training samples for ALL). Each boxplot shows the statistical distribution (minimum, lower quartile, median, upper quartile and maximum) and possible outliers for pure MODIS pixels fully covered by water (blue), soil (orange) and vegetation (green). Every boxplot has the same data cardinality (Table 3 and §2.4); hence, the extent of each box depends only on the data distribution.

In each case, water pixels have the highest NDSI values, as expected from the ANOVA of the field data (Table 4). The median NDSI values for water show that ITA, IND and KHM behave in a similar way, but that VNM has consistently different behaviour from the other three sites, with much higher NDSI. The boxplots show that B4B2 and B1B2 indices from the VIS/NIR family globally confuse water with soil, confirming the field data analysis in which both of them have the lowest separability for the dry soil (DS) condition (Rank: 11 and 12, respectively). The B1B2 index corresponds to NDVI so it is not surprising that it clearly separates vegetation from other categories and that there is overlap between soil and water. The boxplots of B2B6 and B2B7 show a confusion between water and vegetated pixels, again confirming the separability analysis conducted on field data (Rank: 11 and 12, respectively). These results are also in agreement with the post-hoc test on field spectra (Table 4) and confirm the choices of several authors in combining B2B6 with a vegetation index (EVI or NDVI) to reduce this effect. Panel B of Figure 4 provides the results for the combined B2B6-EVI index confirming the capability of this index to separate water for other surfaces. However, when compared to B1B7 we see that the water class is closest to the vegetation class and when data from all sites are grouped, the index values for these categories can overlap. The dotted line on each panel shows the NDSI threshold determined by the application of the recursive partitioning technique to distinguish water from other LC classes and values are reported in Table 7.

**Table 7:** Threshold values (T) for the NDSI/combined index and detection accuracy (%) on pure pixel validation data set.

	VIS/NIR				VIS/SWIR				NIR/NIR	NIR/SWIR		COMBINED
	B4B2	B1B2	B4B5	B1B5	B4B6	B4B7	B1B6	B1B7	B2B5	B2B6	B2B7	B2B6-EVI
**T**	−0.451	−0.470	−0.312	−0.258	−0.228	0.114	−0.223	0.084	0.092	0.271	0.555	0.044
**OA**	87%	81%	95%	91%	**97%**	**96%**	**97%**	**96%**	93%	80%	71%	**97%**
**CE**	19%	26%	4%	6%	2%	2%	3%	3%	3%	20%	28%	**1%**
**OE**	4%	4%	7%	14%	**3%**	7%	**3%**	6%	10%	21%	29%	5%

These thresholds were tested on the validation dataset (500 test pixels per site), and the Overall Accuracy (OA) was computed with the confusion matrices on each NDSI and the combined B2B6-EVI index. We report in Table 7 the results across all four sites. The best mapping accuracy (OA>96%) was obtained for the VIS/SWIR (B4B6, B4B7, B1B6, B1B7) indices and the combined B2B6-EVI index, which agrees with the separability analysis results. Among them, NDSIs with shorter SWIR wavelength (B1B6 and B4B6) have a slightly higher OA (97%) due to a lower Omission Error (OE∼3%). Although OA values are similar (96% and 97%), the Z-test showed that the mapping accuracies obtained with NDSIs that adopt a different SWIR band are statistically different with a confidence level of >95%. Thus, the performance of B1B6 and B4B6 is not significantly different and these indices detect pure water pixels slightly better than B1B7 and B4B7. The blue boxplots in Figure 4 provide an interpretation of the results: the higher OE for B1B7 and B4B7 is mainly caused by low values of NDSI assumed from water pixels in Italy that overlap with the other targets.

The VIS/SWIR indices with a RED band (B1B7 and B1B6) have some skill in separating vegetation and soil pixels compared with the VIS/SWIR indices that use the GREEN band and the combined B2B6-EVI index. The boxplot in Figure 4 shows that the B1B7 and B1B6 indices have higher values in vegetation conditions than B4B6, B4B7 and the combined B2B6-EVI index, in which the boxplots for soil and vegetation are similar. Hence, the Commission Errors (CE) for B1B7 and B1B6 are caused mainly by soil cover, while the CE of B4B7, B4B6 and the combined B2B6-EVI index could result from a confusion across the non-water land cover classes (see also Figure S1 in File S1).

Globally, the analysis of pure MODIS pixels suggests that (i) the indices B1B7 and B4B7 have more skill in separating water pixels from other LCs (Table 6) and that (ii) B1B6 and B4B6 have slightly better performances in detecting pure water pixels (Table 7). Again, the combined B2B6-EVI index is comparable to this category of NDSIs.

### 3.3 The Impact of Different Pixel Mixture Conditions on NDSI Mapping Performance

**Figure 5:**
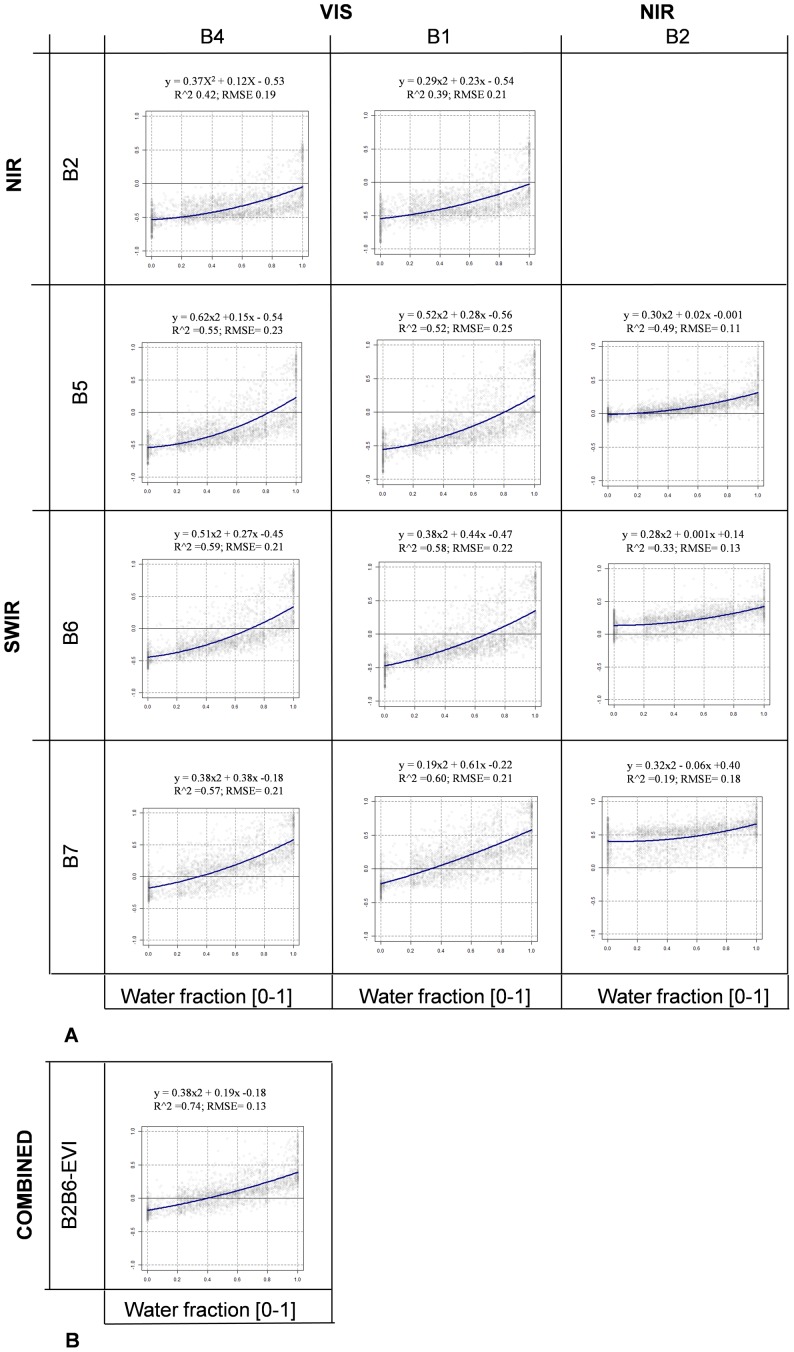
Correlation between the 11 NDSIs and water percentage in MODIS pixels. Panel (a) shows the NDSIs and Panel (b) the combined B2B6-EVI index.

The sensitivity of the NDSIs to water presence was assessed by regression analysis and Noise Equivalent analysis on a data set selected to guarantee equal distribution across sites and classes of water percentage (see §2.4). Figure 5 shows the correlation analysis as a scatterplot matrix, with one plot per NDSI showing the NDSI value on the x axes and the MODIS pixel water percentage on the y axes. Since NDSIs are generally related to water presence in a non-linear manner, we fitted a second-order polynomial regression through the data to better capture saturation behaviour at high water presence. The regression fit and model, across all sites, are shown on each plot and in each case the NDSI value increases more rapidly when the water presence in the MODIS pixel is greater than 70%. Globally, the combined B2B6-EVI index shows the highest correlation with water presence in MODIS pixels (r^2^ = 0.74) followed by B1B7 (r^2^ = 0.60). NDSIs from the family NIR/SWIR (B2B6, B2B7) have the lowest correlation with water presence, r^2^ = 0.33 and 0.19 respectively (See also Figure S2 in File S1 for B2B6-NDVI performance).

Table 8 reports r^2^ and RMSE also for each regression model calculated on data from single sites. Higher correlation is obtained for the VIS/SWIR family of NDSI at all sites, with the coefficient of determination (r^2^) permanently greater than 0.65 and above 0.9 in six cases and above 0.8 in twelve cases. The indices from the VIS/SWIR family outperform the combined B2B6-EVI index in each study area and the minimum r^2^ for the combined index is 0.77 while for B1B7 it is 0.83. This again could indicate that the combined B2B6-EVI index is sensitive to local conditions.

**Table 8:** Regression parameters and performances (r^2^ and RMSE) for the NDSI/combined index vs water fraction relation.

SITE	Coeff.	VIS/NIR				VIS/SWIR				NIR/NIR	NIR/SWIR		COMBINED
		B4B2	B1B2	B4B5	B1B5	B4B6	B4B7	B1B6	B1B7	B2B5	B2B6	B2B7	B2B6-EVI
ITA	intercept	−0.44	−0.42	−0.46	−0.44	−0.41	−0.26	−0.39	−0.25	−0.02	0.05	0.2	−0.21
	x	0.26	0.31	0.21	0.26	0.12	0.03	0.19	0.13	−0.02	−0.16	−0.26	0.13
	x^∧^2	0.01	−0.04	0.31	0.26	0.47	0.64	0.4	0.55	0.28	0.46	0.61	0.37
	r^∧^2	0.54	0.42	0.72	0.65	0.82	0.82	0.77	**0.83**	0.64	0.55	0.36	0.71
	RMSE	0.13	0.18	0.17	0.2	0.14	0.15	0.16	0.15	0.09	0.12	0.2	0.10
IND	Intercept	−0.52	−0.5	−0.56	−0.54	−0.49	−0.28	−0.47	−0.25	−0.06	0.05	0.28	−0.24
	x	0.2	0.13	0.36	0.29	0.54	0.81	0.46	0.73	0.26	0.38	0.45	0.65
	x^∧^2	−0.05	0.02	0.08	0.15	0.02	−0.12	0.09	−0.05	0.05	−0.01	−0.06	−0.14
	r^∧^2	0.58	0.36	0.87	0.78	**0.93**	**0.93**	0.88	**0.93**	0.87	0.72	0.61	0.72
	RMSE	0.08	0.12	0.09	0.12	0.08	0.10	0.10	0.09	0.06	0.10	0.15	0.10
VNM	Intercept	0.45	0.65	0.47	0.73	0.67	0.85	0.96	1.17	−0.12	−0.07	−0.09	−0.17
	x	−0.56	−0.58	−0.54	−0.57	−0.42	−0.1	−0.46	−0.15	0.03	0.20	0.5	0.43
	x^∧^2	0.36	0.27	0.76	0.59	0.48	0.11	0.28	−0.14	0.62	0.45	0.37	0.00
	r^∧^2	0.8	0.81	**0.91**	**0.91**	**0.91**	**0.91**	**0.91**	**0.91**	0.7	0.55	0.46	0.81
	RMSE	0.19	0.21	0.19	0.2	0.17	0.15	0.18	0.16	0.16	0.17	0.16	0.07
KHM	intercept	−0.64	−0.7	−0.64	−0.71	−0.5	−0.09	−0.6	−0.25	−0.01	0.24	0.62	−0.13
	x	−0.07	0.2	0.01	0.27	0.14	0.1	0.52	0.69	0.11	−0.06	−0.27	0.50
	x^∧^2	0.69	0.45	0.77	0.54	0.62	0.55	0.31	0.09	0.09	0.14	0.27	0.29
	r^∧^2	0.69	0.7	0.79	0.79	0.82	**0.87**	0.82	0.86	0.64	0.11	0.03	0.75
	RMSE	0.23	0.23	0.22	0.23	0.19	0.13	0.21	0.16	0.07	0.09	0.10	0.15

**Figure 6:**
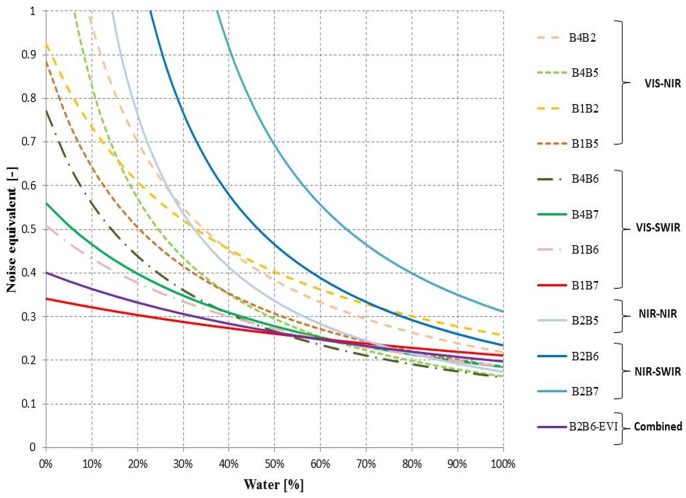
Noise equivalent (see Eq. 4) as a function of water percentage for the 11 NDSIs and the combined B2B6-EVI index.

NE was computed for each NDSI and the results are shown in Figure 6. NE has low values when an NDSI has both high correlation (low RMSE) and high sensitivity (high first derivative) to water presence. Figure 6 shows that the VIS/SWIR NDSI family has the lowest NE (best sensitivity) when water covers less than 50% of the MODIS pixel. Among them, B1B7 has the highest sensitivity to water presence (NE<0.35) when water covers less than 50% of the pixel, followed by B1B6 (NE<0.5) and B4B7 (NE<0.6). The combined B2B6-EVI index is also very sensitive to water presence and is second placed (NE<0.4) after B1B7 (see also Figure S3 in File S1 for B2B6-NDVI performance). The regression and NE analyses both suggest that the B1B7 NDSI is the most sensitive to water presence in mixed land cover MODIS pixels and is comparable to the combined B2B6-EVI index.

The NE results also show that the sensitivity of all indices is comparable (NE<0.3) at high water percentages (>80%) but the NIR/NIR and NIR/SWIR NDSI families lose sensitivity when water presence in MODIS pixels falls below 60%.

### 3.4 Map Accuracy: NDSI Performance Comparison with Pareto Boundary

**Figure 7:**
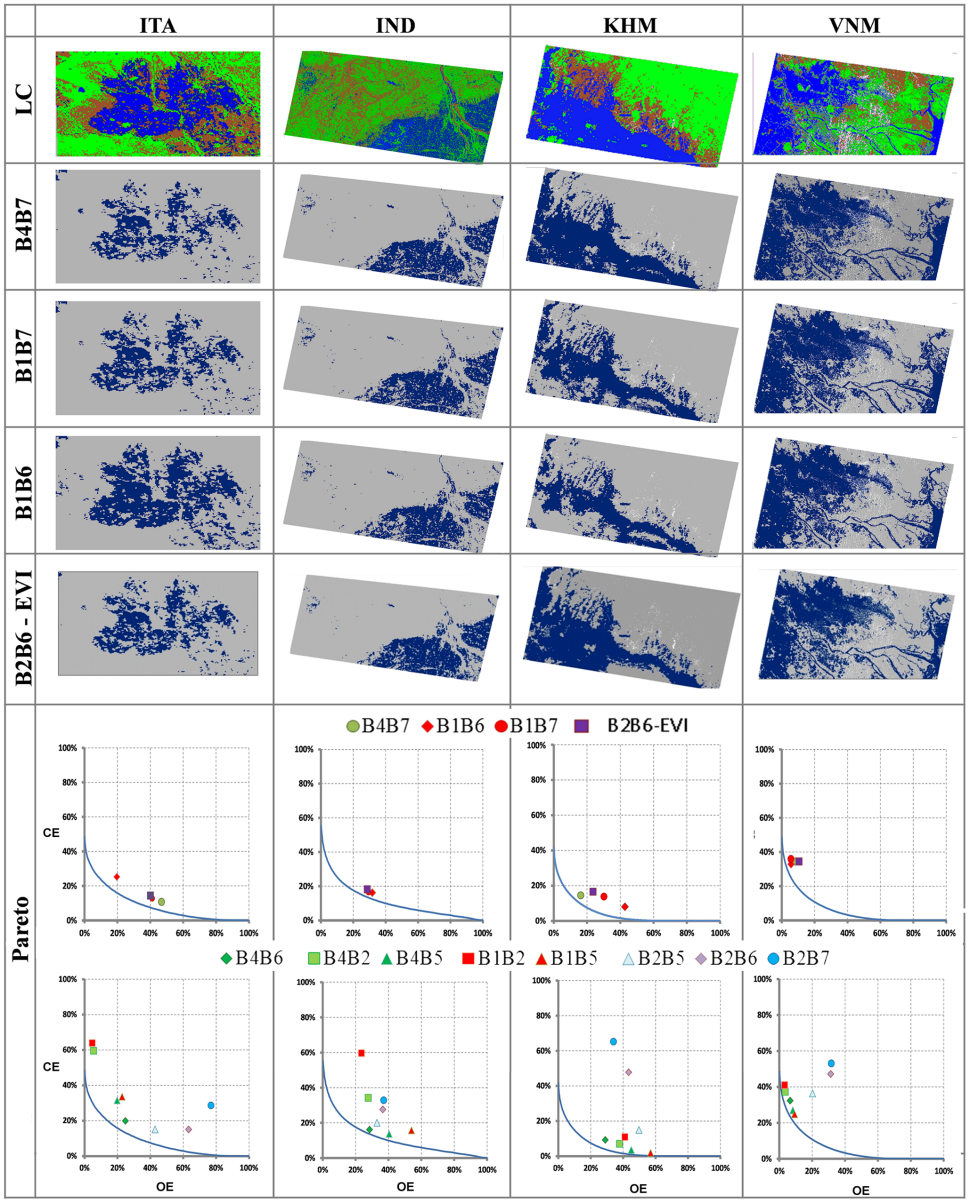
The upper section shows high resolution LC maps (blue: Water; green: vegetation; orange: bare soil; white: clouds) from Landsat analysis and water maps from MODIS for VIS/SVIR indices using global thresholds at the four test sites. The lower section shows the performance of VIS/SWIR, VIS/NIR, NIR/SWIR indices and the combined B2B6-EVI index in the Omission/Commission Error space. Pareto boundaries in the Omission/Commission Error space for each site are provided.

The top section of Figure 7 shows the classified Landsat maps and the results of applying the validated thresholds to the first three NDSIs of the VIS/SWIR family and the combined B2B6-EVI index on the MODIS images across the four sites. These four spectral combinations have consistently outperformed the other NDSIs in the prior analysis steps so we focus on these for reasons of clarity. The lower section of Figure 7 shows the Pareto boundary corresponding to the best possible classification solution for each NDSI in the CE-OE space for each site (see also Figure S4 in File S1 for B2B6-NDVI performance).

The VIS/SWIR family of NDSI is the closest to the Pareto boundaries and never in a region dominated by other NDSIs. In IND and VNM, the mapping accuracy of the VIS/SWIR family of NDSI is comparable (IND: OE∼30% and CE∼20%; VNM: OE∼10% and CE∼35%). At the other two sites, ITA and KHM, the VIS/SWIR indices perform in a different way. In ITA, NDSIs that use SWIR band 7, and the combined B2B6-EVI index, clearly show a higher OE (∼45%) with respect to the VIS/SWIR indices that use band B6 (OE∼25%). In KHM the VIS bands (RED and GREEN) seem to influence the OE: B1B7> B4B7 (∼30% vs. ∼16%) and B1B6> B4B6 (∼43% vs. ∼29%). In this case B4B7 dominates the combined B2B6-EVI index. B1B5 and B4B5 also show good results being close to the Pareto boundary; however, these indices always have the highest omission error. The Pareto boundary plots also show that the IND and ITA sites have a more fragmented landscape (OE up to 80%, CE up to 50%), and hence are more challenging to map with low-resolution data.

Table 9 provides a synthesis of the results for the mapping accuracy on the entire area covered by subsets of the Landsat images. Globally, B4B6 has the highest OA (87%) and first ranking position (1.75), followed by B4B7 (OA 87% and rank 3.75), B1B7 (OA 86% and rank 4.00), B1B6 (OA 85% and rank 4.5) and B4B5 (OA 86% and rank 5.5) but the difference in OA across these five best performing NDSIs is negligible. The combined B2B6-EVI index is again comparable to the VIS/SWIR NDSIs (OA 86% and rank 4.25).

**Table 9:** Overall Accuracy (%) of water mapping for the 11 NDSIs/combined index at the four test sites (IND, ITA, VNM, KHM).

		VIS/NIR				VIS/SWIR				NIR/NIR	NIR/SWIR		COMBINED
	Site/ALL	B4B2	B1B2	B4B5	B1B5	B4B6	B4B7	B1B6	B1B7	B2B5	B2B6	B2B7	B2B6-EVI
OA	IND	85%	70%	89%	86%	**91%**	90%	90%	90%	89%	86%	85%	90%
	ITA	64%	57%	86%	85%	**89%**	87%	88%	88%	87%	83%	79%	88%
	VNM	78%	75%	84%	**86%**	81%	80%	81%	79%	76%	66%	61%	79%
	KHM	84%	82%	83%	79%	87%	**89%**	82%	85%	78%	65%	42%	86%
OA	Value__AVG_	78%	71%	86%	84%	**87%**	**87%**	85%	86%	83%	75%	67%	86%
	Rank__AVG_	8.5	10.5	5.5	6.75	**1.75**	3.75	4.5	4	7.5	9.75	11.25	4.25
OE	Value__AVG_	19%	**18%**	28%	36%	22%	25%	25%	27%	36%	44%	45%	26%
	Rank__AVG_	3.0	**2.5**	8	9.25	4.25	5.75	5.5	5	9.5	10	9.75	5.5
CE	Value__AVG_	35%	44%	**19%**	**19%**	**19%**	**19%**	21%	20%	22%	34%	45%	21%
	Rank__AVG_	8.5	10	**3.5**	**3.5**	4.25	5	4.75	5.5	7.5	8.75	10.5	6.25

Average performance (Value_AVG,_ %) and rank (Rank_AVG_) of OA, OE and CE are provided.

### 3.5 Analysis of Temporal Profiles of NDSIs for MODIS Data

**Figure 8:**
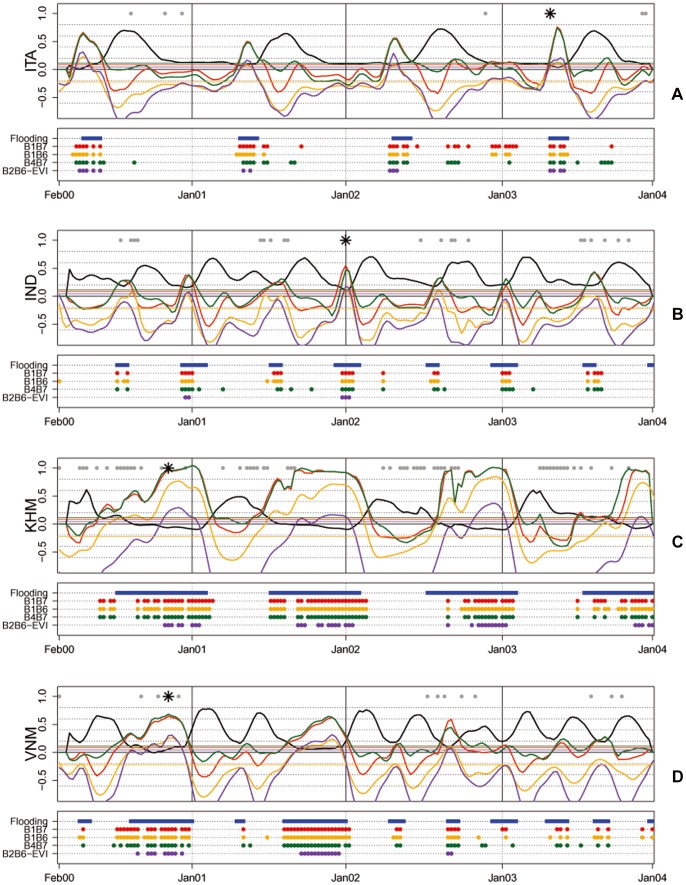
NDSI and combined B2B6-EVI index temporal series for 2000–2003 for four selected rice cultivation areas in Italy (ITA), India (IND), Cambodia (KHM) and Vietnam (VNM). For each site, the top panel shows smoothed profiles of EVI, B1B7, B1B6, B4B7 and B2B6-EVI with black, red, dark green, orange and purple continuous lines respectively. Horizontal lines show the threshold for each index. Grey points indicate cloud contamination, black stars indicate dates of Landsat image acquisition. For each site, the bottom panel shows the flood detection by each index for each MODIS composite date.

Figure 8 shows four years (2000–2003) of temporal series of NDSIs for a single MODIS pixel covering rice production areas at each of the four study sites (Table 3). The same indices used in Figure 7 have been selected to analyse the paddy fields’ temporal signal: B1B7 (red line), B4B7 (dark green line), B1B6 (orange line) and the combined B2B6-EVI index (purple line). EVI (black line) is also reported to describe vegetation growth and to help interpret the agronomic dynamics of the crop. In order to facilitate the temporal interpretation of the NDSI dynamics, the data have been smoothed using a Savitzky–Golay filter weighted for cloud contamination [47]. The continuous coloured lines represent the results of the smoothing procedure which removes the high and low extremes of the original NDSI signals, thus reducing the residual noise due to potential contamination that was not completely eliminated by the MODIS composite algorithm.

Lowland rice is characterised, and can therefore be detected when vegetation growth follows a period of controlled flooding [14]. All the described situations show that agronomic flooding is clearly visible when the indices (NDSI or combined) values surpass the previously derived thresholds reported in the figure as horizontal continuous lines. Flood detection for the indices are reported for each site in the bottom panel where coloured points represents flood detection for each MODIS composite date. This water detection can be interpreted as agronomic flooding because it is followed by an increase in EVI values during the expected crop calendar. Besides this general feature, it is important to note how the MODIS NDSIs are able to identify and characterise different cropping systems. Italy and Cambodia show only one crop season, albeit with different seasonality at each site.

At the European site (ITA, Lat 45°), rice is cultivated in summer with a spring flooding (April–May). At this site, B1B7 and B4B7 identified flood conditions also during the senescent part of the crop cycle and again in December 2002–January 2003 (together with B1B6) when there is permanent water in the field in winter due to heavy rainfall. In Cambodia (KHM, Lat 13°) the season occurs much earlier in spring after a very long period of flood (October–January). The four indices perform in a similar way, detecting flooding in the same periods. The India site (IND, Lat 82°) has a double-season rice cropping system with short-duration varieties, and rice growing is preceded by a short period of flooding which is detected by all four indices. Finally, Vietnam shows triple rice cultivation with three occurrences of flooding followed by a rapid increase in and senescence of EVI, typical of the intensive cultivation of short-duration rice varieties under irrigated conditions. It is interesting to note that the Vietnam site (VNM, Lat 10°) shows a double season in 2000 and 2001 and a triple season in 2002–2003. Moreover, the winter crop is preceded by an intense flooding period whilst the middle-season flooding is less evident in the NDSI signal. This analysis of the multi-year temporal profiles of real MODIS data confirms the results obtained in mapping water on a single date: when using moderate satellite observations, VIS/SWIR indices (B1B7, B4B7, B1B6, and B4B6- data not shown) provide a diagnostic indication of water presence in flooded rice fields. In general these indices have a similar behaviour and water detection across the different indices and thresholds varies only slightly. One index can have a better performance than another in connection with specific conditions of landscape heterogeneity, water thickness and percentage of water presence in the MODIS pixel.

The four indices provide similar values in connection with the SWIR band used, although NDSIs using B7 are more sensitive to water presence. Moreover, the use of the RED band (B1) provides additional information to distinguish bare soil from vegetated conditions. The red and orange lines (Figure 8) show the highest amplitude and have strong drops in connection with maximum vegetation growth shown by the EVI black lines. A flat behaviour of the indices in bare soil conditions can be seen only at the Italian site where fields have no vegetative cover during the winter.

In general, the combined B2B6-EVI index detects flooding less frequently than any of the selected best performing NDSIs. This is particularly evident in IND and VNM where some flooding events are not detected at all. In the IND case study one season is missed in 2000, 2001 and 2002 and both seasons in 2003. Similarly in VNM one season is not detected in 2001 and 2002 and all the three seasons are missing in 2003. These results were obtained using a global threshold value (T_global_ 0.045), however when an optimum local threshold value is calculated for each case study (T_ITA_ = −0.1255; T_IND_ = 0.186, T_KHM_ = −0.22 and T_VNM_ = −0.243) all the agronomic flooding events were detected.

We also tested thresholds available in literature, Xiao et al. [14] and Peng et al [15] respectively equal to −0.05 and −0.21 for each site. These values detect all rice flooding events in ITA, IND and KHM, however in VNM case study the second season – April/May - in 2002 is missed by both and the −0.05 thresholds also misses one season in 2001 and all seasons in 2003.

## Conclusions

In this study, we have analysed and compared a range of NDSIs for detecting surface water in flooded rice fields with the primary aim to identify the most robust strategy, using either the best index or a combination of several indices, that could be used as part of a rice mapping and monitoring system. From a remote sensing point of view, paddy rice flood conditions can be physically interpreted as a shallow water body where soil bottom characteristics strongly influence the reflectance in the VIS-NIR domain. For this reason, we tested NDSIs proposed in the literature specifically for water body detection (e.g. NDWI or MDPI, see Table 1) and/or developed for water content monitoring but also adopted in standing water monitoring. Finally we compared these NDSIs with a combined index (LSWI-EVI equal to B2B6-EVI) usually adopted for rice flood monitoring (see Table 1).

We first classified the proposed NDSIs into different categories in relation to the spectral band used in their calculation, and we added other possible combinations of spectral bands which used the RED and long wavelength SWIR band (2090–2350 nm). The groups showed similar behaviour in relation to the physical processes that govern the targets’ reflection/absorption features in the different spectral domains considered. The NIR-SWIR (800 vs. 1640/2130 nm) and NIR-NIR (800 vs. 1240) indices are proposed in the literature to estimate and monitor plant water content, and in our experimental case they behaved coherently with those assumptions: the higher the NDSI value, the greater the water presence up to completely flooded conditions. On the contrary, the VIS-NIR (550/670 vs. 1240 nm) and VIS-SWIR (550/670 vs. 1640/2130 nm) indices are diagnostic mainly for the detection of water/non-water conditions [21], [48].

The analysis of field spectral data helped to interpret the physical basis behind the band combinations for the various indices, and the ANOVA of the experimental data revealed that all the indices, except for B2B7, provide values that are significantly different for water with respect to the other classes. Specific thresholds for each NDSI were derived from a calibration data set of pure MODIS pixels (water and no water) and their performance was validated on independent data. VIS/SWIR indices outperform the other category, showing OA greater than 96%. The NE analysis showed that the B1B7 index was more sensitive than other NDSIs in low water percentage conditions, but, when water presence exceeds 60%, B4B6, B1B6 and B4B7 slightly outperform B1B7 due to a saturation effect of the B1–B7 combination at high water presence percentages. Very similar results were obtained with the combined B2B6-EVI index.

NDSI water maps at MODIS spatial resolution were validated with Landsat water/no-water data using the Pareto boundary method to account for potential maximum accuracy in relation to low-resolution bias. Results demonstrate again that VIS/SWIR indices are the best and that, in the experimental case, the indices that use B4 bands (B4B6 and B4B7) perform slightly better. The combined B2B6-EVI index also performed well.

The analysis of four years of temporal MODIS data helped visualise the NDSI response throughout the year and the consistency in detection across years. It also confirmed the flood detection results from the single date mapping. All the analysed VIS/SWIR indices detected agronomic flooding of the rice crop except for a few particular events. The combined B2B6-EVI index performed less well with three different global thresholds, and only matched the performance of the VIS/SWIR NDSI when local thresholds were used. From both an information theory view point and an operational detection system viewpoint, a single NDSI (two bands) and a global threshold is preferable to a combined NDSI (four bands when EVI used and three bands when NDVI is use) and a locally adaptive threshold. The small number of sites for the temporal analysis means that this observation should be tested further and we provide some indication of future research directions below.

In conclusion, the VIS/SWIR indices outperform the other NDSIs in water detection with low-resolution data where mixed pixel problems can strongly affect results. Results show that one combination of VIS (GREEN and RED) and SWIR (B6 and B7) bands can outperform another depending on the specific conditions of landscape heterogeneity, water thickness and percentage of water presence in the MODIS pixel. In general NDSIs that use band 7 are much more sensitive to water presence but produce slightly more Commission Error when compared with the ones that use band 6. Therefore, the choice of which VIS/SWIR index to use depends on the aim of water mapping. For rice monitoring, flood mapping is a prerequisite necessary to identify the crop. If adopting a method like the one proposed by [16], [17], crop vegetation growth must follow flood detection. In this framework, an NDSI with high sensitivity to water presence (i.e. less prone to omission) is preferable because the other algorithm criteria (a rapid increase in biomass post-flood) will delete false positive detections. On the other hand, if a more conservative water map is required, NDSIs from the VIS/SWIR family that use the B6 band are suggested.

Rice-growing environments and management systems are diverse, and a robust agronomic flood detection could be developed using a combination of the best-performing NDSIs or combined ones like B2B6-EVI instead of trying to identify one superior NDSI. Stroppiana et al. [49] and Bordogna et al. [50] provided a formal framework which exploits the redundancy and complementary information provided by different spectral combinations, and this method could be adopted as part of an agronomic flood detection. The authors demonstrate the method for burn area mapping in which multiple indices are shown to provide consistent and coherent information over the target of interest as well as complementary, incoherent behaviour over confusing surfaces. Following this approach, a robust agronomic flood mapping method could first use the more conservative indices to select *seed pixels* which have a greater probability to detect only flooded fields (thus reducing false positive error) and then expand the flood detection to the neighbouring pixels with a pixel-growing algorithm on the basis of the results of less conservative indices. Further studies should be conducted to evaluate the feasibility of this kind of approach, for example, testing how positive and negative information provided by a set of NDSIs derived from operational multispectral sensors can be exploited to assess and revise the integrated evidence of agronomic flood in a rice cropping system.

Finally, we have relied on MODIS and Landsat data for our analysis, but, for an operational system, SPOT-VGT data, or the forthcoming PROBA-V with the same spectral characteristics (www.esa.int/SPECIALS/Proba/SEM9FS4PVFG_0.html, accessed September 2012), can also be exploited using a combination of RED B2 (610–680 nm) and MIR (1580–1750 nm) bands. In the future, new opportunities will be provided by the GMES-ESA Sentinel mission (www.esa.int/esaLP/SEMM4T4KXMF_LPgmes_0.html, accessed September 2012). The pair of optical Sentinel-2 satellites, with the first satellite planned for launch in 2014, will routinely deliver high-resolution optical images with VIS-NIR and SWIR bands (1600 and 2200 nm) at 10 and 20 m spatial resolution, respectively. The revisit time of 5 days at the equator and 2–3 days at mid-latitudes would permit rice monitoring in fragmented environments characterised by small fields.

## Supporting Information

File S1 **B2B6-NDVI analysis.** Comments and figures with results for the combined B2B6-NDVI index compared to B2B6-EVI and B1B7.
(DOCX)
