# Supplementary material for: Comparative Analysis of Normalised Difference Spectral Indices Derived from MODIS for Detecting Surface Water in Flooded Rice Cropping Systems
Source: PLoS One. 2014 Feb 20;9(2):e88741. doi: 10.1371/journal.pone.0088741 (PMC3930609; doi:10.1371/journal.pone.0088741)
Supplement: File S1 — B2B6-NDVI analysis. Comments and figures with results for the combined B2B6-NDVI index compared to B2B6-EVI and B1B7. (DOCX) [file pone.0088741.s001.docx]

**Supporting Information: B2B6-NDVI analysis**

B2B6-NDVI has also been used in the literature in combination with B2B6-EVI to detect flooding in rice systems or alone for standing water detection in semi-arid region. We show here the corresponding results for B2B6-NDVI and compare it to B2B6-EVI and B1B7.

The four figures below are additional panels to Figure 4, 5, 6 and 7 in the manuscript. B2B6-NDVI has similar performance to B2B6-EVI in the ability to discriminate between water and other surfaces with a much larger threshold (Figure S1), but has a much lower R^2^ (0.49) in the scatterplot of B2B6-NDVI against water fraction (Figure S2). This demonstrate that only one threshold value for both the combination is not the best solution.

The noise equivalent analysis shows that B2B6-NDVI performs better than other indices with a comparable NE trend to both B2B6-EVI and B1B7. NDVI responds very well to the difference between vegetation and dark background (i.e. water) up to complete canopy cover, after which it can saturate and present more noise (see vertical scatter at high water fractions for B2B6-NDVI in the Figure S2).

The overall mapping performance of B2B6-NDVI, using a specific threshold derived in this study by the available data set, are comparable to the other two index. Evident differences can be appreciate by the analysis of OE and CO in the Pareto space. In the ITA case study B2B6-NDVI produces a higher CE with a reduce OE respect the other two indices. In the KHM site B2B6-NDVI have an opposite behaviour with higher OE. Finally in VNM B2B6-NDVI and B1B7 slightly dominate B2B6-EVI.

**Figure S1 (additional panel for Figure 4): Boxplots of B1B7 vs combined B2B6-EVI/NDVI indices for pure water pixels (blue), pure soil pixels (orange) and pure vegetation pixels (green). Every boxplot has the same cardinality. The vertical dotted lines show the calculated threshold.**

| Band combination | B1B7 | B2B6-EVI | B2B6-NDVI |
| --- | --- | --- | --- |
| Threshold | 0.084 | 0.045 | -0.208 |
| Box plots of index value | 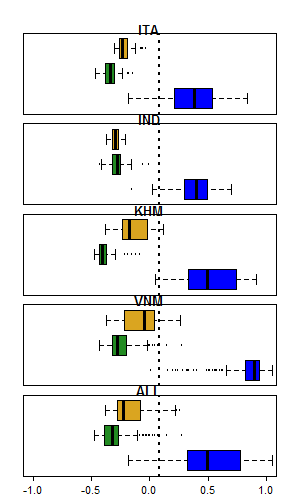 | 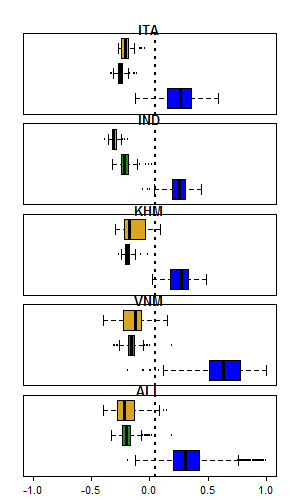 | **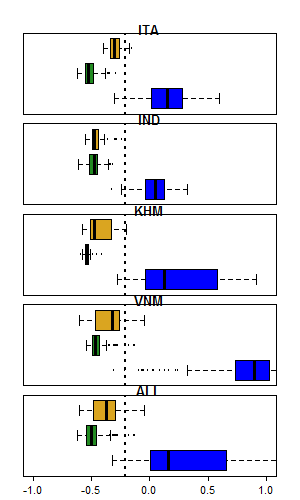** |

**Figure S2 (additional panel for Figure 5): Correlation between the B1B7, B2B6-EVI and B2B6-NDVI and water percentage in MODIS pixels.**

| Band combination | B1B7 | B2B6-EVI | B2B6-NDVI |
| --- | --- | --- | --- |
| Threshold | 0.084 | 0.045 | -0.208 |
| Index |  |  | **** |
|  | Water fraction [0-1] | Water fraction [0-1] | Water fraction [0-1] |

**Figure S3 (additional panel for Figure 6): Noise equivalent (see Eq. 4 in manuscript) as a function of water percentage for the B1B7 and the combined indices B2B6-EVI and B2B6-NDVI.**

**Figure S4 (additional panel for Figure 7): performance of B1B7 and the combined indices B2B6-EVI and B2B6-NDVI in the Omission/Commission Error space for each site. Blue line refers to Pareto boundaries.**

|  | **ITA** |  | **IND** |
| --- | --- | --- | --- |
| **Pareto** |  | **Pareto** |  |
| **OA** | B1B7 88%  B2BE-EVI 88%  B2B6-NDVI 88% | **OA** | B1B7 90%  B2BE-EVI 90%  B2B6-NDVI 90% |
|  |  |  |  |
|  | **KHM** |  | **VNM** |
| **Pareto** |  | **Pareto** |  |
| **OA** | B1B7 85%  B2BE-EVI 86%  B2B6-NDVI 83% | **OA** | B1B7 79%  B2BE-EVI 79%  B2B6-NDVI 80% |

B1B7 B2B6-EVI B2B6-NDVI
